# Associations between novel anthropometric indices and the prevalence of gallstones among 6,848 adults: a cross-sectional study

**DOI:** 10.3389/fnut.2024.1428488

**Published:** 2024-07-22

**Authors:** Jie Zhang, Depeng Liang, Lidong Xu, Yanhong Liu, Shan Jiang, Xiaomeng Han, Huili Wu, Yuanyuan Jiang

**Affiliations:** ^1^Department of Gastroenterology, Zhengzhou Central Hospital Affiliated to Zhengzhou University, Zhengzhou, China; ^2^Henan Provincial Medicine Key Laboratory of Colorectal Cancer Diagnosis and Treatment, Zhengzhou, China; ^3^Zhengzhou Key Laboratory of Colorectal Cancer Diagnosis, Treatment and Research, Zhengzhou, China

**Keywords:** novel anthropometric indices, gallstones, cross-sectional study, abdominal obesity, NHANES (National Health and Nutrition Examination Survey)

## Abstract

**Background:**

Traditional anthropometric measures, including body mass index (BMI), are insufficient for evaluating gallstone risk. This study investigated the association between novel anthropometric indices and gallstone risk among 6,848 participants from the National Health and Nutrition Examination Survey in the United States.

**Methods:**

Measures calculated included weight (WT), BMI, waist circumference (WC), waist-to-height ratio (WtHR), conicity index (CI), A Body Shape Index (ABSI), Body Roundness Index (BRI), Abdominal Volume Index (AVI), and Weight-adjusted Waist Index (WWI). Logistic regression and smooth curve fitting assessed the relationships between these indices and gallstones, complemented by receiver operating characteristic (ROC) curve analysis to evaluate their discriminative power.

**Results:**

The results indicated significant differences between study groups, with a positive and independent correlation identified between gallstones and all measures except ABSI. Specifically, per 1 SD increase in WC, WT, BMI, WtHR, and AVI was associated with a 57%, 59%, 52%, 53%, and 53% increased risk of gallstones, respectively. Dose-response analysis confirmed a positive correlation between these indices and gallstone risk. ROC analysis highlighted WtHR and BRI as having superior discriminative abilities (AUC = 0.6703). Further, among participants with a BMI < 30 kg/m2, elevated levels of WT, WtHR, CI, BRI, and WWI significantly increased the risk of gallstones (*P* < 0.001). Likewise, elevated BMI heightened the risk at low levels of WT, WC, WtHR, BRI, AVI, and CI (*P* < 0.001).

**Conclusion:**

This study supports the positive association between various anthropometric indicators and gallstones, recommending that newer anthropometric indices be considered more extensively to enhance gallstone prevention and treatment strategies.

## 1 Introduction

Gallstones, one of the most prevalent digestive system disorders in the world, cause symptoms including nausea, vomiting, lack of appetite, and discomfort in the abdomen and stomach (1). Gallstones, also known as cholelithiasis, are defined by the buildup of sediments in the gallbladder or common bile duct that are made of mineral or fatty deposits. Additionally, gallstones have been linked to higher rates of occurrence and death from common chronic conditions such as diabetes, hypertension, cardiovascular disease, and gastrointestinal tumors (2–6). Gallstones are estimated to afflict 10–15% of the population in America and other affluent nations and up to 70% of American Indians, according to epidemiologic data (7, 8). In America, gallstones bring about a significant health care burden (9). Numerous risk variables, including race, gender, and age above 40, are linked to the development of gallstones (10, 11). Other risk factors include metabolic syndrome, rapid weight loss, medication use, and a family history of gallstones. Patients are at a significant risk of obesity because of the metabolic alterations brought on by obesity. These changes include abnormalities in the liver and gallbladder, among other metabolic organs, as well as hyperlipidemia, impaired intestinal motility, and increased bile secretion in the liver (12). Type 2 diabetes mellitus, obesity, insulin resistance, and nonalcoholic fatty liver disease (NAFLD) are all regarded as elements of the metabolic syndrome. The most significant risk factor for both gallbladder stones and metabolic syndrome is abdominal fat. It is associated with increased synthesis of cholesterol within the body, excessive bile cholesterol secretion, and a rise in bile-related gallstone-causing components. Studies have shown an obvious correlation between obesity and the development of gallstones, particularly obesity in the abdomen (10). Moreover, for every 5 units increase in body mass index (BMI), the incidence of gallstones has increased 1.63 times (13).

To separate the distribution of body fat, a few novel anthropometric indices (AHIs) have been introduced in the last few decades to measure obesity, particularly central obesity. Anthropometric indices are simple metrics for evaluating nutritional health and rapidly determining illness risk (14). Examples of AHIs include body mass index (BMI), waist circumference (WC), curve index (CI), body roundness index (BRI), abdominal volume index (AVI), weight-adjusted waist index (WWI), and waist-to-height ratio (WtHR). ABSI has been proven to be associated with mortality, diabetes, hypertension, and metabolic syndrome (5, 6). Body fat percentage and visceral fat can be predicted using the body roundness index (BRI) (4). According to Gaoteng Lin’s research, there is a correlation between the risk of kidney stones and the innovative AHIs that completely reflect an individual’s body shape. These findings can be crucial in determining the risk of kidney stones (KS) (15).

On the other hand, our understanding of how well these indicators predict gallstone risk is somewhat limited. It is still difficult to determine the best anthropometric index for gallstone screening. The purpose of this study is to evaluate and compare the nine anthropometric indices (AVI, CI, WWI, WtHR, BMI, WT, WC, ABSI, BRI, and WtHR) as screening measures for gallstone risk in American adults. The ideal cutoff values for these indices enable medical professionals and health policy makers to estimate the gallstone risk. Subsequently, risk reduction interventions can target high-risk participants, thereby reducing the risk of developing gallstones.

## 2 Materials and methods

### 2.1 Study design and participants

This study included baseline data from the cycles of the National Health and Nutrition Examination (NHANES). In order to investigate the prevalence of health, nutrition, and possible risk factors, the National Health and Nutrition Examination Survey (NHANES) conducted a series of interviews and physical examinations on a nationally representative sample of the US population each 2 years. In the study, the data for participants aged over 20 years old who participated in the gallstone questionnaire was extracted from NHANES cycles conducted between 2017 and 2020, and the question was: “Have you ever had gallstones?” Our study received an exemption from the Institutional Review Board as all participants gave written informed permissions in the initial survey and their personal data was completely de-identified. A total of 15,560 individuals completed the questionnaire. The following were the exclusion criteria (Supplementary Figure 1). Ultimately, this study composed 6,848 patients in total, including 720 self-reported gallstone history.

### 2.2 Anthropometric index calculation

Expert examiners used established methods and tools at the mobile examination center to measure fundamental anthropometric metrics such as waist circumference, height, and body weight. Using previously published formulas, compute BMI, ABSI, BRI, AVI, CI, WWI and WtHR. As follows:

BMI = WT (kg) / height^2^(m)

ABSI = WC (cm) / [BMI^2/3^(kg/m2) × height^1/2^(m)]

BRI = 364.2−365.5 × {1− [(WC (cm) / 2π) / (0.5 × height(m))]^2^ }^0.5^

AVI = {2 × (WC^2^ (cm)) + 0.7 × (WC (cm)-Hip(cm))^2^} / 1000

CI = WC(m) / 0.109/(Weight(kg) / Height(cm))^0.5^

WWI = WC (cm) / Weight^0.5^(cm/kg^0.5^)

WtHR = WC (cm) / Height(cm)

### 2.3 Ascertainment of other covariates

The interview identified age, gender (male/female), and race/ethnicity (Mexican American, Other Hispanic, Non-Hispanic White, Non-Hispanic Black, and Other Race). Education level is divided into high school graduates or below, partial university graduates, and university graduates or above. Marital status is categorized as Married/Living with a Partner, Divorced/Separated/Widowed, and Never Married. The poverty ratio was categorized as the ratio of monthly family income to poverty levels and categorized into 3 groups: <1.3 (low income), 1.3–3.5 (middle income), > 3.5 (high income), and missing. Physical activities such as walking or cycling, employment, and amusement are all considered sports. It is considered an active activity when a participates in one or more of those activities. It will be regarded as a non-active activity otherwise. Total calorie consumption, total water intake, total carbohydrate intake, total sugar intake, total protein intake, and total fat intake are dietary characteristics. A 2-day average of the intake was used for quantification. Serum was tested for Uric acid (umol/L), serum cholesterol (mg/dl), Sodium (mmol/L), Iron (umol/L), Chloride (mmol/L), Bicarbonate (mmol/L), Potassium (mmol/L). A history of CVD is a previous diagnosis of heart failure, coronary heart disease, angina, heart attack, or stroke. The classification of smoking is as follows: current smoking (smoking ≥ 100 cigarettes and current smokers), previous smoking (smoking ≥ 100 cigarettes but not currently smoking), never smoking (never smoking or smoking ≤ 100 cigarettes). Had 12 or more drinks of alcohol in the previous year? (yes/no). A blood pressure inspector who has completed an approved training program takes the patient’s blood pressure. The standardized blood pressure of each participant was determined by averaging the three measurements. Having a systolic blood pressure of at least 140 mmHg and/or a diastolic blood pressure of at least 90 mmHg, or being on antihypertensive medication, was the definition of hypertension. One of the following criteria was used to diagnose diabetes mellitus: a) self-reported diabetes; b) fasting blood glucose ≥ 126 mg/dl; c) HBA1c level ≥ 6.5%; and d) usage of anti-diabetic medications, such as insulin. A cancer history was established based on the self-report of the NHANES medical condition questionnaire.

### 2.4 Statistical analysis

Because NHANES uses a complex multi-stage probability sampling design method to represent a nationally representative large sample. Therefore, we use linear regression analysis to summarize continuous variables as mean values with standard error (SE). 95% confidence intervals and weighted survey averages are used to describe continuous variables, while categorical variables are described by weighted survey averages and 95% confidence intervals. In the study, all anthropometric variables performed z score conversion as follows: z score = (index-index_mean_) / index_sd_ (Supplementary Table 1). Through weighted multivariate logistic regression analysis. In model 1, no adjustments were made. In model 2, adjusted for gender, age, race, and marital status. In model 3, adjusted for all variables and used a smooth curve fitting to analyze the nonlinear relationship between anthropometric indices and the incidence of gallstones. Examine the effects of heterogeneity with a subgroup analysis. Assess the ability to discriminate of several anthropometric indices for gallstone patients by comparing the area under the curve (AUC) and the ROC curve. The DeLong’s test is used to evaluate statistical differences between AUCs. Body mass index is classified into two categories based on suggestions set out by the World Health Organization (WHO). A body mass index of > 30 kg/m2 is considered obese. Other anthropometric indicators are bisected based on the best cutoff point in ROC analysis. We further investigated to improve the assessment of gallstone risk by combining BMI with other anthropometric measures. Use Spearman method to perform correlation analysis on two types of anthropometric measurements. Statistical significance was defined as a *p-*value < 0.05. All the analyses were conducted using R version 4.0.2 (The R Foundation)^1^ and Empower software (X&Y Solutions, Inc., Boston, MA, USA)^2^.

## 3 Results

### 3.1 Baseline characteristics

Table 1 shows the baseline characteristics of NHANES cohort participants with complete information on gallstones and anthropometric indicators. In NHANES from 2017 to 2020, a total of 6848 participants with complete information on gallstones were analyzed after combining anthropometric indicators and covariates. A total of 6128 people (89.49%) had no history of gallstones, while 720 people (10.51%) had a history of gallstones. We found that in the study population, patients with gallstones had almost all higher anthropometric indicators than those without gallstones. It’s interesting to note that there was not an apparent difference in ABSI between the two groups. In the gallstone group, the proportion of alcohol consumption and active exercise was lower than in the normal group (*P* < 0.05), and the age of onset, proportion of women, proportion of hypertension, diabetes, cancer, and CVD history were all significantly higher than in the normal group. Interestingly, the total fat intake of non-gallstone patients is higher than that of gallstone patients.

**TABLE 1:** Baseline characteristics of participants, weighted.

	Nonstone formers (*n* = 6128)	Stone formers (*n* = 720)	*P-*value
Age, years	47.22 (45.99, 48.46)	56.76 (55.28, 58.24)	<0.001
Total water intake, gm	2943.43 (2882.26, 3004.59)	2780.28 (2647.26, 2913.31)	0.031
Total energy intake, gm	2112.25 (2083.83, 2140.67)	1926.69 (1858.16, 1995.22)	<0.001
Total protein intake, gm	81.63 (80.18, 83.08)	71.76 (68.69, 74.83)	<0.001
Total carbohydrate intake, gm	238.50 (233.94, 243.05)	225.64 (215.29, 236.00)	0.040
Total Sugar intake, gm	101.18 (97.59, 104.77)	103.14 (96.47, 109.81)	0.630
Total Fat intake, gm	86.78 (85.63, 87.94)	80.69 (76.39, 84.99)	0.009
Serum calcium, mg/dL	9.29 (9.27, 9.32)	9.29 (9.21, 9.36)	0.776
Serum phosphorus, mg/dL	3.58 (3.56, 3.61)	3.59 (3.52, 3.67)	0.780
Creatinine, umol/L	78.06 (77.16, 78.96)	76.26 (73.92, 78.60)	0.141
Serum Cholesterol, mg/dl	187.37 (185.10, 189.63)	188.48 (183.77, 193.20)	0.584
HDL-Cholesterol, mg/dL	53.76 (52.90, 54.62)	52.94 (51.49, 54.38)	0.315
Triglyceride, mg/dL	109.04 (104.51, 113.56)	119.19 (107.97, 130.40)	0.070
LDL-Cholesterol, mg/dL	109.73 (107.38, 112.07)	110.68(105.22, 116.14)	0.721
BMI	29.39 (29.05, 29.73)	32.93 (32.05, 33.82)	<0.001
WT	83.82 (82.73, 84.90)	89.61 (86.77, 92.45)	<0.001
WC	99.96(90.00, 100.93)	108.00(105.79, 110.21)	<0.001
WtHR	0.59 (0.59, 0.60)	0.66 (0.64, 0.67)	<0.001
BRI	5.48 (5.35, 5.61)	7.00 (6.69, 7.31)	<0.001
ABSI	0.08 (0.08, 0.08)	0.08 (0.08, 0.08)	<0.001
AVI	20.63 (20.23, 21.03)	24.07 (23.11, 25.02)	<0.001
WWI	10.98 (10.93, 11.03)	11.49 (11.39, 11.58)	<0.001
CI	1.31 (1.30, 1.31)	1.35 (1.34, 1.36)	<0.001
Uric acid, umol/L	318.35 (314.96, 321.74)	322.47 (312.36, 332.57)	0.444
Serum Sodium, mmol/L	140.47 (139.95, 141.00)	140.25 (139.62, 140.87)	0.054
serum Iron, umol/L	16.05(15.74, 16.36)	14.87(14.28, 15.47)	0.001
serum Chloride, mmol/L,	101.28 (100.99, 101.56)	100.96 (100.59, 101.32)	0.021
serum Bicarbonate, mmol/L	25.58 (25.34, 25.82)	25.56 (25.13, 25.99)	0.927
serum Potassium, mmol/L	4.11 (4.07, 4.15)	4.09 (4.04, 4.13)	0.085
GENDER, *N* (%)			<0.001
male	51.88 (49.99, 53.77)	26.79 (22.82, 31.18)	
female	48.12 (46.23, 50.01)	73.21 (68.82, 77.18)	
PIR, *N* (%)			0.003
<1.3	16.29 (14.77, 17.94)	15.67 (11.72, 20.64)	
1.3–3.5	30.10 (27.36, 32.98)	39.61 (32.30, 47.42)	
≥3.5	43.41 (40.22, 46.66)	36.56 (31.99, 41.39)	
missing	10.20 (8.66, 11.97)	8.16 (5.73, 11.49)	
Race/ethnicity, *N* (%)			0.015
Mexican American	8.28 (6.23, 10.93)	7.25 (5.06, 10.28)	
Other Hispanic	7.26 (5.89, 8.90)	7.21 (4.63, 11.07)	
Non-Hispanic White	64.28 (59.02, 69.22)	70.77 (64.05, 76.70)	
Non-Hispanic Black	10.93 (8.28, 14.31)	6.60 (4.87, 8.87)	
Other Race	9.25 (7.50, 11.35)	8.17 (5.44, 12.09)	
Education levels, *N* (%)			0.109
High school or less	37.03 (33.68, 40.52)	40.06 (35.09, 45.25)	
Some college or associates degree	30.43 (28.36, 32.59)	33.47 (28.25, 39.14)	
College graduate or above	32.53 (28.35, 37.02)	26.47 (20.09, 34.01)	
Marital status, *N* (%)			0.004
Married/Living with Partner	61.97 (59.17, 64.69)	64.27 (58.40, 69.74)	
Widowed/Divorced/Separated	17.89 (16.44, 19.44)	23.11 (18.91, 27.92)	
Never married	20.14 (17.94, 22.54)	12.62 (9.41, 16.71)	
Alcohol, *N* (%)			<0.001
No	37.79 (34.83, 40.85)	52.18 (46.49, 57.82)	
Yes	54.64 (51.60, 57.64)	38.38 (33.63, 43.36)	
Missing	7.57 (6.59, 8.68)	9.44 (6.98, 12.65)	
Physical activity, *N* (%)			<0.001
Inactive	18.21 (16.77, 19.74)	27.74 (23.40, 32.54)	
Active	81.79 (80.26, 83.23)	72.26 (67.46, 76.60)	
Smoking, *N* (%)			0.041
Never	57.16 (55.14, 59.16)	52.24 (45.89, 58.51)	
Former	25.76 (23.84, 27.77)	32.03 (26.69, 37.89)	
Current	17.08 (14.89, 19.52)	15.73 (12.41, 19.74)	
Hypertension, *N* (%)			<0.001
No	62.72 (59.88, 65.48)	43.59 (38.26, 49.07)	
Yes	37.28 (34.52, 40.12)	56.41 (50.93, 61.74)	
Diabetes (%), *N* (%)			<0.001
No	86.30 (85.39, 87.16)	73.29 (68.79, 77.36)	
Yes	13.70 (12.84, 14.61)	26.71 (22.64, 31.21)	
Cancer, *N* (%)			<0.001
No	89.75 (88.79, 90.64)	82.47 (77.30, 86.66)	
Yes	10.25 (9.36, 11.21)	17.53 (13.34, 22.70)	
CVD, *N* (%)			<0.001
No	91.35 (89.53, 92.88)	83.46 (80.08, 86.36)	
Yes	8.65 (7.12, 10.47)	16.54 (13.64, 19.92)	

Data of continuous variables: survey-weighted mean (*95%CI*), *P-value* was by survey-weighted linear regression. Data of categorical variables: survey-weighted percentage (*95%CI*), *P-value* was by survey-weighted Chi-square test. HDL-Cholesterol, High Density Lipoprotein Cholesterol; LDL-Cholesterol, Low Density Lipoproteins Cholesterol; ABSI, A Body Shape Index; BMI, body mass index; CI, conicity index; WC, waist circumference; WT, weight; WtHR, waist-to-height ratio; BRI, body roundness index; AVI, abdominal volume index; WWI, weight-adjusted waist index; PIR, poverty income ratio; CVD, cardiovascular disease.

### 3.2 Associations between nine anthropometric measures and gallstones

**FIGURE 1:**
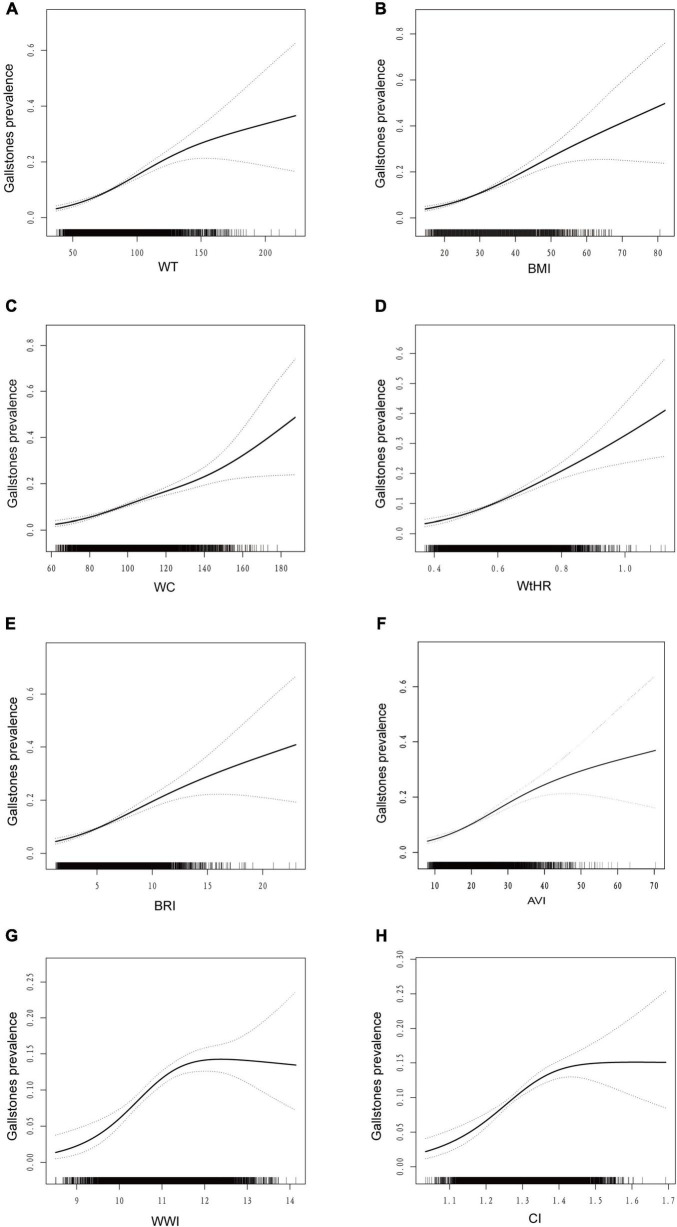
Dose–response relationship analysis between anthropometric measures and gallstones. The area between the upper and lower dashed lines is represented as the 95% CI. Each point shows the magnitude of the anthropometric measures and is connected to form a continuous line. GAM regression was adjusted for gender, age, race/ethnicity, marital status, physical activity, alcohol, smoking, total water intake, total carbohydrate intake, total fat intake, total protein intake, hypertension, diabetes, cancer, CVD. **(A)** association between WT and the risk of gallstones; **(B)** association between BMI and the risk of gallstones; **(C)** association between WC and the risk of gallstones; **(D)** association between WtHR and the risk of gallstones; **(E)** association between BRI and the risk of gallstones; **(F)** association between AVI and the risk of gallstones; **(G)** association between WWI and the risk of gallstones; **(H)** association between CI and the risk of gallstones. BMI, body mass index; CI, conicity index; WC, waist circumference; WT, weight; WtHR, waist-to-height ratio; BRI, body roundness index; AVI, abdominal volume index; WWI, weight-adjusted waist index.

Almost all anthropometric indicators are positively correlated with gallstones (Table 2). In model 1, WWI had the highest OR (per 1 SD increment) (OR: 1.90; 95%CI: 1.68–2.16, *P* < 0.001) across all anthropometric indicators. After adjusting for covariates of age, gender, race, marital status, physical activity, smoking, alcohol, hypertension, diabetes, cancer, CVD history, Total water intake, Total carbohydrate intake, Total Fat intake, Total protein intake, WtHR (OR: 1.53; 95%CI: 1.36–1.71; *P* < 0.001), BRI (OR: 1.48; 95%CI: 1.33–1.65; *P* < 0.001), AVI (OR: 1.53; 95%CI: 1.38–1.70; *P* < 0.001), WWI (OR: 1.29; 95%CI: 1.11–1.49; *P* < 0.001), CI (OR: 1.34; 95%CI: 1.17–1.54; *P* < 0.001), WT (OR: 1.59; 95%CI: 1.43–1.75; *P* < 0.001),WC (OR: 1.57; 95%CI: 1.40–1.76; *P* < 0.001)and BMI (OR: 1.52; 95%CI: 1.39–1.67; *P* < 0.001) were still associated with gallstones in model 3. Furthermore, in models 2 and 3, there was no apparent association between the prevalence of gallstones and ABSI. An additive generalized model and smoothed curve fitting were applied to examine the association between gallstones prevalence and anthropometric indicators (Figure 1).

**TABLE 2:** Logistic regression analysis of anthropometric indices and gallstones.

	Model 1		Model 2		Model 3	
	OR (95% CI)	*P*-value	OR (95% CI)	*P*-value	OR (95% CI)	*P*-value
WC Z-score	1.56 (1.40, 1.74)	<0.001	1.65 (1.47, 1.86)	<0.001	1.57 (1.40, 1.76)	<0.001
WtHR Z-score	1.79 (1.60, 2.00)	<0.001	1.62 (1.44, 1.82)	<0.001	1.53 (1.36, 1.71)	<0.001
BRI Z-score	1.71 (1.55, 1.89)	<0.001	1.57 (1.42, 1.74)	<0.001	1.48 (1.33, 1.65)	<0.001
ABSI Z-score	1.35 (1.21, 1.50)	<0.001	1.05 (0.93, 1.18)	0.468	0.98 (0.87, 1.10)	0.766
AVI Z-score	1.52 (1.37, 1.67)	<0.001	1.61 (1.44, 1.79)	<0.001	1.53 (1.38, 1.70)	<0.001
WWI Z-score	1.90 (1.68, 2.16)	<0.001	1.42 (1.23, 1.65)	<0.001	1.29 (1.11, 1.49)	<0.001
CI Z-score	1.68 (1.48, 1.91)	<0.001	1.48 (1.28, 1.70)	<0.001	1.34 (1.17, 1.54)	<0.001
WT Z-score	1.27(1.15, 1.41)	<0.001	1.65 (1.48, 1.83)	<0.001	1.59 (1.43, 1.75)	<0.001
BMI Z- score	1.55(1.42, 1.68)	<0.001	1.59 (1.46, 1.74)	<0.001	1.52 (1.39, 1.67)	<0.001

Model 1: unadjusted model. Model 2: gender, age, Race/ethnicity, Marital status. Model 3: gender, age, Race/ethnicity, Marital status, Physical Activity, Alcohol, Smoking, Total water intake, Total carbohydrate intake, Total Fat intake, Total protein intake, Hypertension, Diabetes, Cancer, CVD. ABSI, A Body Shape Index; BMI, body mass index; CI, conicity index; WC, waist circumference; WT, weight; WtHR, waist-to-height ratio; BRI, body roundness index; AVI, abdominal volume index; WWI, weight-adjusted waist index.

**FIGURE 2:**
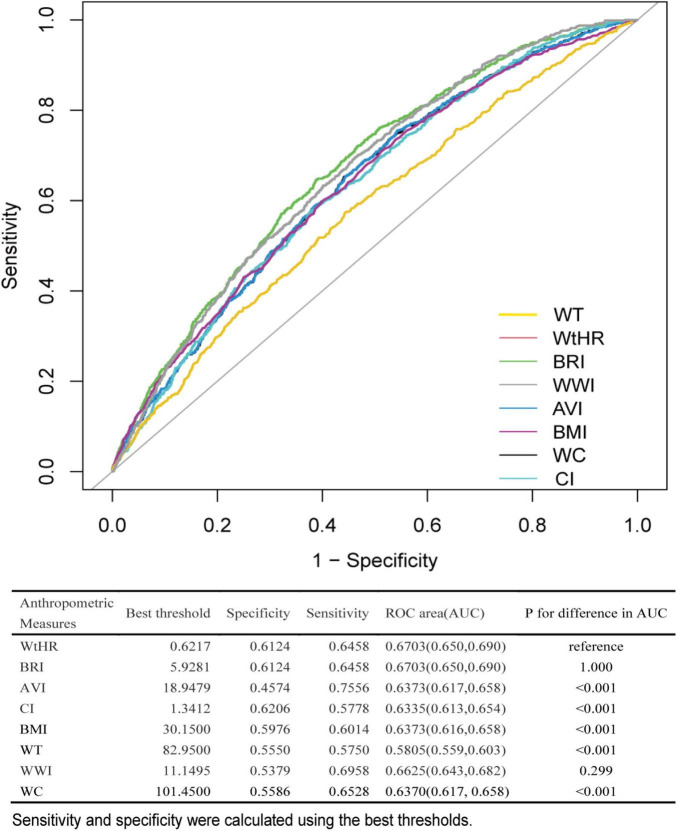
ROC curves of anthropometric measures for discriminating gallstone. ROC, receiver operating characteristic; AUC, area under the curve; BMI, body mass index; CI, conicity index; WC, waist circumference; WT, weight; WtHR, waist-to-height ratio; BRI, body roundness index; AVI, abdominal volume index; WWI, weight-adjusted waist index.

In Figure 2, we found there is a nonlinear association between WWI and CI indicators and gallstone prevalence, while WT, WC, BMI, AVI, WtHR, and BRI are linearly and positively associated with gallstone prevalence. We also carried out subgroup analyses (Supplementary Table 2), stratified by age (<60 and ≥60 years), sex (male and female) and BMI (<30 and ≥30 kg/m2). The subjects were divided by age (≥60 and <60 years), gender (male and female), and BMI (≥30 and <30 kg/m2). The results indicate a correlation between anthropometric indicators and gallstones, however, for individuals under 60 years old, females, and those with a BMI < 30 kg/m2, most anthropometric indicators show a stronger correlation with gallstones.

### 3.3 Discrimination ability of different anthropometric measures

**FIGURE 3:**
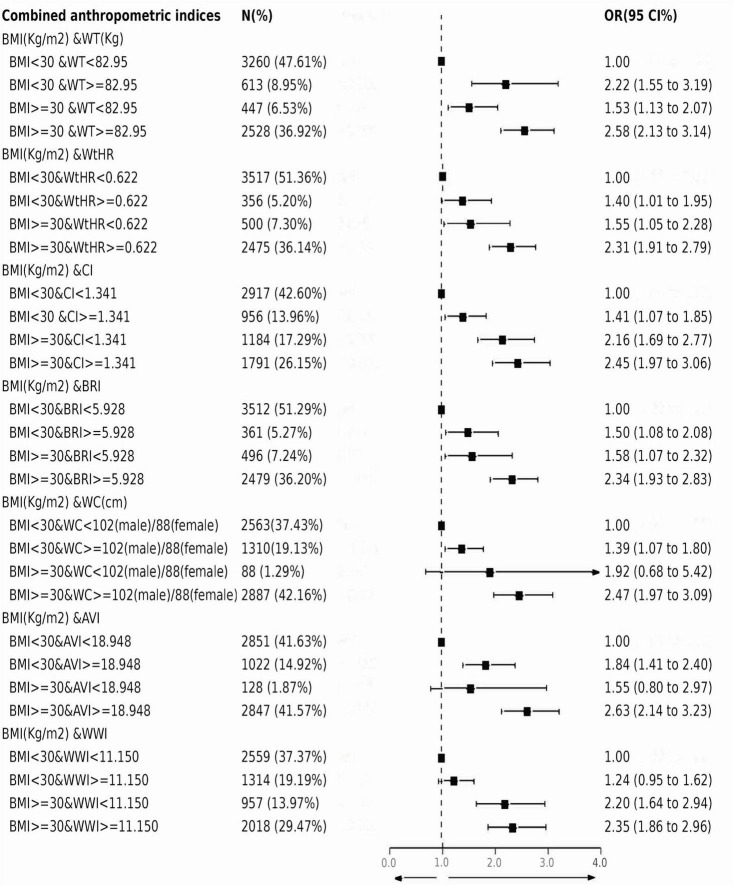
Association between gallstones and combined anthropometric indices. Multivariable logistic regression was conducted after adjusting for confounding factors of gender, age, race/ethnicity, marital status, physical activity, alcohol, smoking, total water intake, total carbohydrate intake, total fat intake, total protein intake, hypertension, diabetes, cancer, CVD.

The abilities of several anthropometric indicators to distinguish people with gallstones were assessed using receiver operating characteristic curves (ROC curves) and area under the curve (AUC) (Figure 3). Compared to the other 6 anthropometric indicators, the results show that WtHR and BRI had the best diagnostic abilities (WtHR: AUC = 0.670 95%CI:0.650–0.690; BRI: AUC = 0.670 95%CI: 0.650–0.690). We find WWI, AVI, WC, BMI, CI, and WT all showed favorable AUC values. Furthermore, stratified by BMI ( < 30 and ≥ 30 kg/m2), subgroup ROC curve studies were also performed. When comparing participants with a BMI ≥ 30 kg/m2, we discovered that WtHR and BRI had the highest discriminating power. Participants with a BMI < 30 kg/m2 had comparatively higher AUC values for all 7 anthropometric indicators (Supplementary Table 3).

### 3.4 Combination of BMI and other anthropometric indices

The Spearman method was used to analyze the correlation between several anthropometric indicators. The correlation with BMI was the strongest for BRI (*r* = 0.921). While CI showed a minimal correlated with BMI (*r* = 0.503) (Supplementary Table 4). Since BMI is the most commonly used body measure, we evaluated the risk of gallstones in this study by combining BMI with other body measures. As shown in Figure 3, individuals with a BMI < 30 kg/m2 showed positive associations between elevated WT (OR: 2.22; 95%CI: 1.55–3.19; *P* < 0.001),WC (OR: 1.39; 95%CI: 1.07–1.80; *P* = 0.014), WtHR (OR: 1.40; 95%CI: 1.01–1.95; *P* = 0.045),CI (OR: 1.41; 95%CI: 1.07–1.85; *P* = 0.013), BRI (OR: 1.50; 95%CI: 1.08–2.08; *P* = 0.014), AVI (OR: 1.84; 95%CI: 1.41–2.40; *P* < 0.001) and gallstone incidence. However, participants with a BMI < 30 kg/m2 but increased WWI did not show an increased risk of gallstones (*P* > 0.05). In participants with normal WT, WtHR, CI, BRI and WWI, an elevated BMI also increased the risk of gallstones (all *P* < 0.05). Gallstone risk significantly increased with higher BMI and other anthropometric indices. The odds ratios (ORs) for WT, WC, WtHR, CI, BRI, AVI, and WWI were 2.58 (95%CI: 2.13–3.14), 2.47 (95%CI: 1.97–3.09), 2.31 (95%CI: 1.91–2.79), 2.45 (95%CI: 1.97–3.06), 2.34 (95%CI: 1.93–2.83), 2.63 (95%CI: 2.14–3.23), and 2.35 (95%CI: 1.86–2.96), respectively. Additionally, participants with a BMI ≥ 30 kg/m2 along with other abnormal anthropometric indicators had the highest risk of gallstones.

### 3.5 Sensitivity analysis

In sensitivity analysis (Supplementary Table 5), we excluded extreme values with anthropometric measures greater than 99% or less than 1%. The results were consistent with the main analysis results, showing that all 8 anthropometric indicators were positively correlated with the incidence of gallstones. WC (OR: 1.54; 95%CI: 1.33–1.77; *P* < 0.001), WtHR (OR: 1.50; 95%CI: 1.30–1.72; *P* < 0.001), BRI (OR: 1.48; 95%CI: 1.29–1.68; *P* < 0.001), AVI (OR: 1.52; 95%CI: 1.33–1.75; *P* < 0.001), WWI (OR: 1.30; 95%CI: 1.12–1.52; *P* < 0.001), CI (OR: 1.30; 95%CI: 1.11–1.52; *P* < 0.001), WT (OR: 1.59; 95%CI: 1.40–1.82; *P* < 0.001), and BMI (OR: 1.54; 95%CI: 1.36–1.73; *P* < 0.001). And we excluded participants with hematological disorders, finding the results to remain reliable (Supplementary Table 6).

## 4 Discussion

Our research is the first comprehensive examination of the relationship between gallstones and anthropometric indices within a sizable, nationally representative cohort of the American population. This study, based on two cycles (2017–2020) of the NHANES database, investigated eight anthropometric indices (WtHR, BRI, AVI, WWI, CI, WC, BMI, and WT). In the fully adjusted model, these indices showed a positive correlation with the risk of gallstones. Of these, BRI demonstrated the most significant discriminating power. Additionally, WtHR, with its simpler computation, offers discriminative capacity comparable to BRI.

Metabolic syndrome (MetS) and gallstone disease share common risk factors, including insulin resistance and central obesity (16, 17). Studies identify obesity as a distinct risk factor for gallstones. Although metabolic surgery has emerged as an effective tool for sustainable weight loss and glycemic control, gallstone formation before and after bariatric surgery will continue to concern clinicians (18). A recent study by Khalid O. Alyahyawi et al. found a causal relationship between gallstone disease, total bilirubin levels, and BMI (19). According to many studies, an increase in BMI is an independent risk factor for the occurrence of gallstones (20, 21). The chance of gallstone development increases by 1.63 times for every 5 percentage point increase in BMI (13). Interestingly, we also found that this association is more pronounced in women than in men (22). Obese individuals, regardless of metabolic health, are more likely to have gallstones than healthier counterparts, suggesting that obesity can independently accelerate gallstone formation. Interestingly, the total fat intake of the gallstone group was lower than that of the non-gallstone group, which may be related to the higher proportion of women in the gallstone group and their generally older age. The results of Wei et al.’s study are consistent with those of this study (23). Previous studies have shown that the increased risk of gallstones is associated with high intake of energy, total fat, saturated fatty acids, and monounsaturated fatty acids (24). However, in Tsai et al.’s study, excessive intake of unsaturated fats may reduce the risk of male gallstones (25). Mendelian randomization studies have shown that unsaturated fatty acids reduce the risk of cholecystitis and gallstones (26). Meanwhile, obese individuals have a higher risk of cholesterol stone formation compared to normal weight individuals in terms of gallbladder peristalsis (27). These findings highlight that whether a patient has metabolic syndrome or not, they can still benefit from maintaining a normal weight to prevent gallstones (28). Increased hepatic bile secretion, inadequate bile acid secretion, and increased liver absorption and use of cholesterol are all linked to hyperinsulinemia. Additional research has demonstrated an independent association between gallstones, type 2 diabetes, and insulin resistance. Higher insulin levels observed in gallstone patients suggest that insulin resistance is a significant risk factor for the formation of gallstones (29). According to research by Méndez Sánchez et al., individuals with gallstones had insulin levels that were 26.2% higher (OR*:* 2.3; 95%CI 1.14–4.66, *p* = 0.03) than those in the control group (30).

Currently, most research uses BMI and weight as the primary indicators of obesity. However, in young, muscular individuals, what may appear as obesity could actually be the result of increased muscle mass (31). Additionally, BMI may not accurately quantify obesity in older adults due to the decrease in muscle mass (32). However, weight and BMI cannot accurately reflect the distribution of body fat (33, 34). To examine various obesity patterns more precisely, several novel anthropometric techniques have been introduced recently. These techniques are specifically designed to differentiate between central obesity and the emergence of chronic illnesses.

Research shows that ABSI is an independent predictor of all-cause mortality rates (35). There is a significant correlation between visceral fat, cardiovascular disease, and ABSI. Visceral fat, which may be used to indicate arterial stiffness in T2DM patients, showed a significant correlation with ABSI in a cross-sectional study of individuals with the disease (36). According to a systematic study, ABSI was more accurate in predicting all-cause mortality than both body mass index (BMI) and waist circumference (WC), though it was less accurate in predicting chronic illnesses. Like BMI and WC, excessive central obesity is linked to an increased risk of chronic illnesses. However, unlike these measures, ABSI does not differentiate between fat and lean mass. Due to its high degree of clustering around the mean and low variation, ABSI’s predictability may be limited. While ABSI outperforms BMI and WC in predicting all-cause mortality, it is less effective in predicting chronic illnesses (37). This study also found no correlation between ABSI and gallstones after adjusting for confounding factors.

The World Health Organization (WHO) suggests measuring the circumference of the abdomen at the point where the top edge of the ilium and the lower edge of the ribs meet to determine the precise measurement technique for the WtHR. According to a meta-analysis, WtHR is a better predictor of diabetes, hypertension, and cardiovascular diseases than WC and BMI (38). In a study conducted in China, elevated BMI, WC, and WtHR were identified as independent risk factors for newly diagnosed gallstones (20). The model used to determine the Body Roundness Index (BRI) is based on a theoretically derived constant. Comparisons of BRI with conventional measures such as BMI, weight, or height have shown improved predictions of body fat percentage and visceral fat index (39). Additionally, elevated BRI levels have been independently associated with T2DM episodes, according to prior research. BRI is also directly linked to organ damage caused by hypertension, including left ventricular hypertrophy, microalbuminuria, arterial stiffness, and lower limb atherosclerosis (40). Moreover, CI has been used to diagnose T2DM and hypertension. According to Mantzoros et al., CI was not only a predictor of hypertension risk but also correlated with lipid profiles and fasting blood insulin levels (41). Research also indicates a link between the AVI and several conditions, including metabolic syndrome, diabetes, nonalcoholic fatty liver disease, hypertension, and declining renal function (42–45). However, the relationships between the BRI, CI, and AVI with the risk of gallstones remain unclear. The WWI is a new obesity assessment metric that normalizes WC to body weight, offering a simpler and more rational approach than BMI alone (46). Previous studies have shown that WWI is positively correlated with mortality rates of hypertension, diabetes, and cardiovascular disease (47, 48). In a study conducted in the United States, a higher WWI was also found to be positively correlated with the incidence of gallstones (49). This aligns with the outcomes that WWI demonstrated in our study. Our analysis shows that WtHR and BRI have a more significant impact on the incidence of gallstones compared to other central obesity markers. Based on the ROC curve analysis, WtHR and BRI also exhibited superior diagnostic capacity relative to other anthropometric measurements, offering a reliable forecast for gallstone risk. The study has several advantages and implications. Firstly, it is based on a substantial sample size that provides robust statistical power. Secondly, we utilized smooth curve fitting to illustrate the varying incidence rates of obesity levels and the nonlinear relationships between gallstones and different measurement techniques. Thirdly, anthropometric indicators proved more effective than conventional measurements in screening for gallstone risk, highlighting a strong link between abdominal obesity and gallstones. Anthropometric markers present a simple, non-invasive method for gallstone risk screening, which is beneficial from a public health perspective. However, the study also faces significant limitations. The primary limitation of our study is that the NHANES data on gallstones, derived from patient questionnaire surveys without conducting imaging studies, which may have bias. Secondly, as a cross-sectional study, it cannot establish causal relationships. Therefore, it is imperative for a well-designed prospective study to further investigate the impact of novel anthropometric indices on the incidence of gallstone, and for our observations to be validated. Thirdly, the absence of data on fat content and distribution precludes a direct evaluation of the relationship between body measurements and body or visceral fat. Fourthly, since the NHANES study focuses on the non-institutionalized U.S. population, the results may not be generalizable to other populations.

## 5 Conclusion

In this cross-sectional study, anthropometric indices including WT, WC, BMI, WtHR, BRI, AVI, WWI, and CI, were found to have a significant positive association with gallstones. Specifically, WT (per 1 SD increment) exhibited the strongest association with gallstones, while WtHR and BRI demonstrated superior discriminatory power for detecting gallstone risk. Assessing the risk of gallstones with BMI alone is insufficient. Greater consideration should be given to recently established anthropometric indicators to enhance gallstone prevention and treatment.

## Data availability statement

The datasets presented in this study can be found in online repositories. The names of the repository/repositories and accession number(s) can be found in this article/Supplementary material.

## Ethics statement

The studies involving humans were approved by the NCHS Research Ethics Review Committee reviewed and approved the NHANES study protocol. The studies were conducted in accordance with the local legislation and institutional requirements. The participants provided their written informed consent to participate in this study.

## Author contributions

JZ: Writing – review and editing, Writing – original draft, Methodology, Conceptualization. DL: Writing – review and editing, Writing – original draft, Methodology, Conceptualization. LX: Writing – review and editing, Supervision, Methodology, Conceptualization. YL: Writing – original draft, Formal analysis, Data curation, Conceptualization. SJ: Writing – original draft, Formal analysis, Data curation, Conceptualization. XH: Writing – original draft, Formal analysis, Data curation, Conceptualization. HW: Writing – review and editing, Writing – original draft, Supervision, Investigation, Funding acquisition, Conceptualization. YJ: Writing – review and editing, Writing – original draft, Supervision, Investigation, Funding acquisition, Conceptualization.

## Footnotes

1
http://www.Rproject.org

2
www.empowerstats.com
