# Supplementary material for: Associations between novel anthropometric indices and the prevalence of gallstones among 6,848 adults: a cross-sectional study
Source: Front Nutr. 2024 Jul 22;11:1428488. doi: 10.3389/fnut.2024.1428488 (PMC11298442; doi:10.3389/fnut.2024.1428488)
Supplement: Supplementary file 1 [file Data_Sheet_1.docx]

Associations between novel anthropometric indices and the prevalence of gallstones among 6,848 adults: A cross-sectional study

Jie Zhang^1,2,3†^, Depeng Liang^1,2,3†^, Lidong Xu^1,2,3^, Yanhong Liu^1,2,3^, Shan Jiang^1,2,3^, Xiaomeng Han^1,2,3^, Huili Wu^1,2,3^* and Yuanyuan Jiang^1,2,3^*

^1^Department of Gastroenterology, Zhengzhou Central Hospital Affiliated to Zhengzhou University, Zhengzhou, 450007, China

^2^Henan Provincial Medicine Key Laboratory of Colorectal Cancer Diagnosis and Treatment, Zhengzhou, 450007, China

^3^Zhengzhou Key Laboratory of Colorectal Cancer Diagnosis, Treatment and Research, Zhengzhou, 450007, China

Figure S1. Flow chart of the screening process for the selection of the study population.


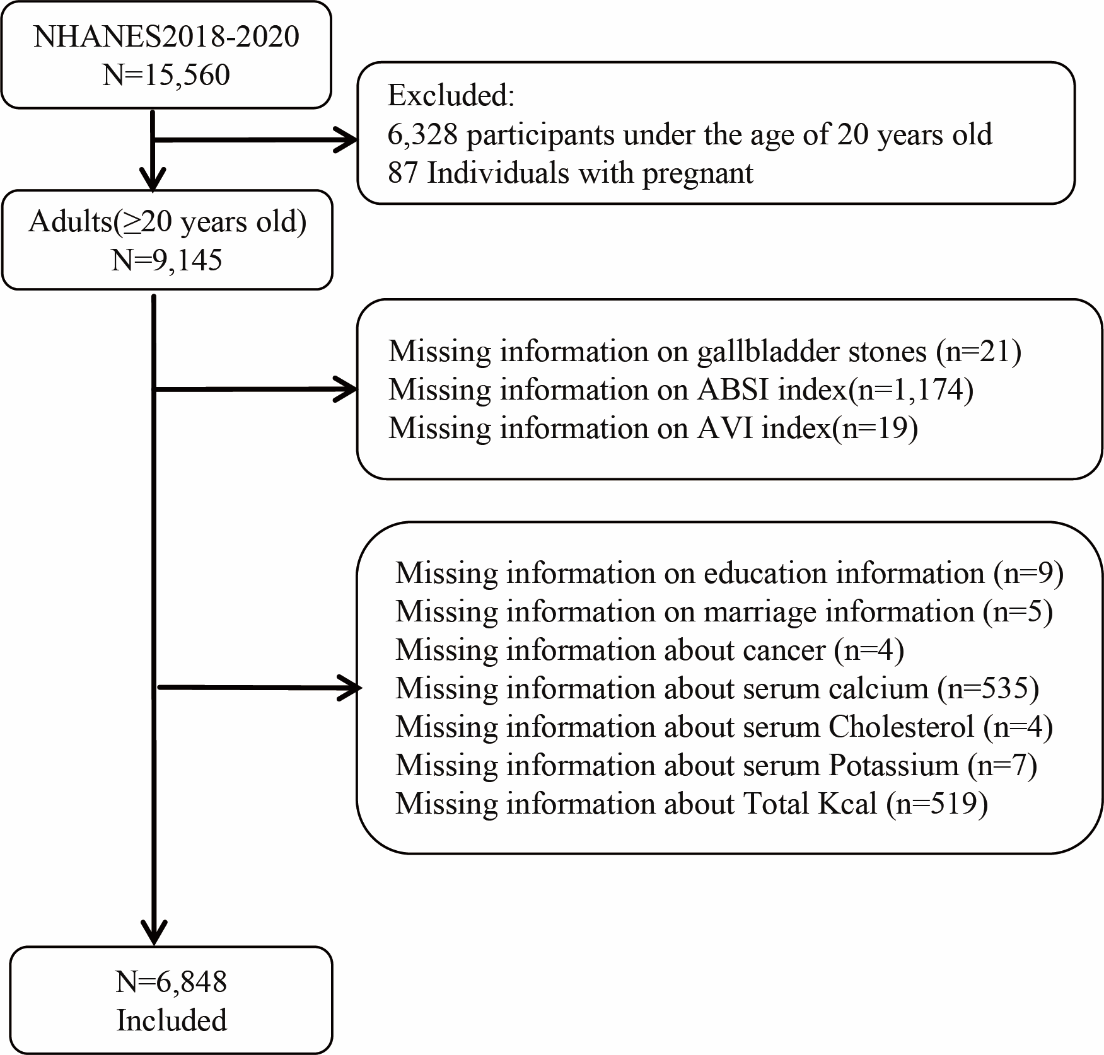


Abbreviations: NHANES, National Health and Nutrition Examination Survey; ABSI, A Body Shape Index; AVI, Abdominal volume index.

**Table S1** The baseline characteristics of novel anthropometric indicators after Z-score transformation, weighted.

|  | Total (n = 6,848) | Non-stone formers (n = 6,128) | Stone formers (n = 720) | P-value |
| --- | --- | --- | --- | --- |
| BMI Z-score | -0.04 (-0.09, 0.01) | -0.09 (-0.14, -0.04) | 0.40 (0.28, 0.52) | <0.001 |
| WT Z-score | 0.02 (-0.03, 0.07) | -0.01 (-0.06, 0.04) | 0.25 (0.12, 0.37) | <0.001 |
| WC Z-score | -0.03 (-0.08, 0.03) | -0.08 (-0.13, -0.02) | 0.39 (0.26, 0.52) | <0.001 |
| WtHR Z-score | -0.07 (-0.12, -0.01) | -0.13 (-0.19, -0.08) | 0.48 (0.35, 0.60) | <0.001 |
| BRI Z-score | 0.28 (0.22, 0.33) | 0.21 (0.16, 0.27) | 0.82 (0.70, 0.95) | <0.001 |
| ABSI Z-score | 0.20 (0.15, 0.26) | 0.18 (0.12, 0.23) | 0.44 (0.34, 0.54) | <0.001 |
| AVI Z-score | -0.02 (-0.08, 0.03) | -0.08 (-0.13, -0.02) | 0.40 (0.27, 0.53) | <0.001 |
| WWI Z-score | -0.09 (-0.15, -0.04) | -0.16 (-0.22, -0.10) | 0.43 (0.32, 0.55) | <0.001 |
| CI Z-score | -0.05 (-0.11, 0.01) | -0.11 (-0.17, -0.04) | 0.38 (0.26, 0.49) | <0.001 |

For continuous variables: survey-weighted mean (95%CI), P-value was by survey-weighted linear regression (svyglm). Abbreviations: BMI, body mass index; WT, weight; WC, waist circumference; WtHR, waist-to-height ratio; BRI, body roundness index; ABSI, A Body Shape Index; AVI, abdominal volume index; WWI, weight-adjusted waist index; CI, conicity index.

**Table S2** Subgroups analysis

|  | Age | | Sex | | BMI | |
| --- | --- | --- | --- | --- | --- | --- |
|  | ≥60 years (n=2436) | <60 years (n=4412) | Males (n=3373) | Females (n=3475) | ≥30 kg/m^2^ (n=2975) | <30 kg/m^2^ (n=3873) |
| WC Z-score | 1.31 (1.04, 1.65) | 1.68(1.43, 1.97) | 1.33 (1.04, 1.70) | 1.64 (1.45, 1.86) | 1.58 (1.33, 1.88) | 1.99 (1.49, 2.64) |
| P for interaction | 0.114 | | 0.108 | | 0.163 | |
| WtHR Z-score | 1.25 (1.00, 1.56) | 1.68 (1.42, 1.97) | 1.37 (1.09, 1.72) | 1.57 (1.40, 1.77) | 1.57(1.30, 1.89) | 1.78(1.35, 2.34) |
| P for interaction | 0.056 | | 0.223 | | 0.459 | |
| BRI Z-score | 1.23 (1.00, 1.52) | 1.61 (1.38, 1.87) | 1.35 (1.08, 1.68) | 1.52 (1.36, 1.69) | 1.46 (1.26, 1.69) | 1.88 (1.39, 2.54) |
| P for interaction | 0.067 | | 0.294 | | 0.134 | |
| AVI Z-score | 1.29 (1.03, 1.61) | 1.62 (1.39, 1.88) | 1.30 (1.04, 1.63) | 1.60 (1.43, 1.80) | 1.48 (1.28, 1.71) | 2.17 (1.58, 2.99) |
| P for interaction | 0.136 | | 0.086 | | 0.030 | |
| WWI Z-score | 1.16 (0.96, 1.39) | 1.49 (1.25, 1.79) | 1.44(1.18, 1.76) | 1.33 (1.15, 1.52) | 1.21 (1.01, 1.44) | 1.29 (1.08, 1.54) |
| P for interaction | 0.057 | | 0.491 | | 0.643 | |
| CI Z-score | 1.23 (1.01, 1.49) | 1.51 (1.26, 1.81) | 1.42(1.12, 1.79) | 1.39(1.20, 1.60) | 1.38 (1.16, 1.64) | 1.22 (1.03, 1.44) |
| P for interaction | 0.143 | | 0.866 | | 0.301 | |
| BMI Z-score | 1.20 (0.98, 1.49) | 1.62 (1.42, 1.84) | 1.26(0.99, 1.61) | 1.55(1.41, 1.70) | / | / |
| P for interaction | 0.038^***^ | | 0.116 | | / | |
| WT Z-score | 1.28 (1.03, 1.60) | 1.63 (1.43, 1.85) | 1.22 (0.94, 1.58) | 1.66(1.49, 1.84) | 1.51 (1.32, 1.73) | 2.07 (1.41, 3.03) |
| P for interaction | 0.105 | | 0.038 | | 0.128 | |

When analyzing a subgroup variable gender, age, Race/ethnicity, Marital status, Physical Activity, Alcohol, Smoking, Total water intake, Total carbohydrate intake, Total Fat intake, Total protein intake, Hypertension, Diabetes, Cancer, CVD were adjusted except for the variable itself.

Abbreviations: ABSI, A Body Shape Index; BMI, body mass index; CI, conicity index; WC, waist circumference; WT, weight; WtHR, waist-to-height ratio; BRI, body roundness index; AVI, Abdominal volume index; WWI, Weight-adjusted waist index.

Values are OR (95%CI) Pvalue

**Table S3** ROC analyses in BMI subgroup

| Subgroups | Anthropometric Measures | Best threshold | Specificity | Sensitivity | ROC area (AUC) | *P* for difference in AUC |
| --- | --- | --- | --- | --- | --- | --- |
| BMI>=30 |  |  |  |  |  |  |
|  | WtHR | 0.6999 | 0.6291 | 0.5411 | 0.613(0.585,0.641) | Reference |
|  | BRI | 7.8869 | 0.6291 | 0.5411 | 0.613(0.585,0.641) | 1.000 |
|  | AVI | 25.4389 | 0.4773 | 0.6073 | 0.553(0.523,0.582) | <0.001^***^ |
|  | CI | 1.3738 | 0.5684 | 0.5411 | 0.557(0.527,0.586) | <0.001^***^ |
|  | WT | 123.85 | 0.8711 | 0.1644 | 0.498(0.467,0.528) | <0.001^***^ |
|  | WWI | 11.5246 | 0.5479 | 0.6301 | 0.605(0.577,0.633) | *0.489* |
|  | WC | 112.9500 | 0.4848 | 0.6005 | 0.559(0.526,0.588) | <0.001^***^ |
| BMI<30 |  |  |  |  |  |  |
|  | WtHR | 0.5266 | 0.4080 | 0.8227 | 0.661(0.630,0.693) | Reference |
|  | BRI | 3.8706 | 0.4080 | 0.8227 | 0.661(0.630,0.693) | 1.000 |
|  | AVI | 17.1500 | 0.5717 | 0.5957 | 0.616(0.583,0.650) | <0.001^***^ |
|  | CI | 1.2912 | 0.5564 | 0.6702 | 0.651(0.620,0.682) | 0.338 |
|  | WT | 59.2500 | 0.2008 | 0.8333 | 0.501(0.467,0.535) | <0.001^***^ |
|  | WWI | 10.7427 | 0.4812 | 0.773 | 0.669(0.640,0.699) | *0.406* |
|  | WC | 92.3500 | 0.5664 | 0.5993 | 0.623(0.590,0.659) | <0.001^***^ |

^†^Sensitivity and Specificity were calculated using the best thresholds.

Abbreviations: BMI, body mass index; CI, conicity index; WC, waist circumference; WT, weight; WtHR, waist-to-height ratio; BRI, body roundness index; AVI, Abdominal volume index; WWI, Weight-adjusted waist index.

^***^ *P*-value<0.001.

**Table S4** Pearson correlation analysis among different Anthropometric Measures.

|  | WC | WtHR | BRI | AVI | CI | BMI | WT | WWI |
| --- | --- | --- | --- | --- | --- | --- | --- | --- |
| WC | 1.000 | 0.936 | 0.927 | 0.993 | 0.780 | 0.907 | 0.899 | 0.651 |
| WtHR |  | 1.000 | 0.992 | 0.929 | 0.773 | 0.914 | 0.742 | 0.785 |
| BRI |  |  | 1.000 | 0.933 | 0.744 | 0.921 | 0.746 | 0.757 |
| AVI |  |  |  | 1.000 | 0.751 | 0.915 | 0.906 | 0.625 |
| CI |  |  |  |  | 1.000 | 0.503 | 0.466 | 0.921 |
| BMI |  |  |  |  |  | 1.000 | 0.889 | 0.470 |
| WT |  |  |  |  |  |  | 1.000 | 0.260 |
| WWI |  |  |  |  |  |  |  | 1.000 |

Abbreviations: BMI, body mass index; CI, conicity index; WC, waist circumference; WT, weight; WtHR, waist-to-height ratio; BRI, body roundness index; AVI, Abdominal volume index; WWI, Weight-adjusted waist index.

**Table S5** Sensitivity analysis of Anthropometric Measures and gallbladder stone.

|  |  | Model 1 |  | Model 2 |  | Model 3 |  |
| --- | --- | --- | --- | --- | --- | --- | --- |
|  | N | OR (95% CI) | P-value | OR (95% CI) | P-value | OR (95% CI) | P-value |
| WC Z-score | 6714 | 1.56(1.36, 1.79) | <0.001 | 1.64 (1.42, 1.89) | <0.001 | 1.54 (1.33, 1.77) | <0.001 |
| WtHR Z-score | 6712 | 1.79 (1.57, 2.04) | <0.001 | 1.59 (1.39, 1.83) | <0.001 | 1.50 (1.30, 1.72) | <0.001 |
| BRI Z-score | 6712 | 1.75 (1.55, 1.98) | <0.001 | 1.57 (1.38, 1.79) | <0.001 | 1.48 (1.29, 1.68) | <0.001 |
| AVI Z-score | 6712 | 1.54 (1.36, 1.76) | <0.001 | 1.62 (1.41, 1.86) | <0.001 | 1.52 (1.33, 1.75) | <0.001 |
| WWI Z-score | 6712 | 1.93 (1.68, 2.23) | <0.001 | 1.45 (1.24, 1.69) | <0.001 | 1.30 (1.12, 1.52) | <0.001 |
| CI Z-score | 6712 | 1.63 (1.41, 1.89) | <0.001 | 1.43 (1.22, 1.69) | <0.001 | 1.30 (1.11, 1.52) | <0.001 |
| WT Z-score | 6712 | 1.23 (1.09, 1.40) | 0.004 | 1.66 (1.45, 1.91) | <0.001 | 1.59 (1.40, 1.82) | <0.001 |
| BMI Z- score | 6718 | 1.57 (1.40, 1.77) | <0.001 | 1.61 (1.43, 1.82) | <0.001 | 1.54 (1.36, 1.73) | <0.001 |

Model 1: unadjusted model

Model 2: gender, age, Race/ethnicity, Marital status

Model 3: gender, age, Race/ethnicity, Marital status, Physical Activity, Alcohol, Smoking, Total water intake, Total carbohydrate intake, Total Fat intake, Total protein intake, Hypertension, Diabetes, Cancer, CVD.

Abbreviations: BMI, body mass index; CI, conicity index; WC, waist circumference; WT, weight; WtHR, waist-to-height ratio; BRI, body roundness index; AVI, Abdominal volume index; WWI, Weight-adjusted waist index.

**Table S6.** Logistic regression analysis of anthropometric measures and gallbladder stone without hematological disorders.

|  | Model 1 | | Model 2 | | Model 3 | |
| --- | --- | --- | --- | --- | --- | --- |
|  | OR (95% CI) | P-value | OR (95% CI) | P-value | OR (95% CI) | P-value |
| WC Z-score | 1.55 (1.39, 1.73) | <0.001 | 1.65 (1.46, 1.86) | <0.001 | 1.57 (1.21, 2.02) | 0.004 |
| WtHR Z-score | 1.78 (1.60, 1.99) | <0.001 | 1.61 (1.44, 1.81) | <0.001 | 1.52 (1.17, 1.97) | 0.005 |
| BRI Z-score | 1.71 (1.55, 1.89) | <0.001 | 1.57 (1.41, 1.74) | <0.001 | 1.48 (1.17, 1.87) | 0.005 |
| ABSI Z-score | 1.34 (1.20, 1.49) | <0.001 | 1.04 (0.92, 1.17) | 0.476 | 0.97 (0.75, 1.25) | 0.704 |
| AVI Z-score | 1.51 (1.37, 1.67) | <0.001 | 1.60 (1.44, 1.78) | <0.001 | 1.53 (1.21, 1.92) | 0.004 |
| WWI Z-score | 1.90 (1.67, 2.15) | <0.001 | 1.42 (1.22, 1.65) | <0.001 | 1.28 (0.93, 1.76) | 0.041 |
| CI Z-score | 1.67 (1.47, 1.90) | <0.001 | 1.47 (1.28, 1.69) | <0.001 | 1.34 (0.99, 1.81) | 0.024 |
| WT Z-score | 1.27(1.14, 1.40) | <0.001 | 1.64 (1.46, 1.84) | <0.001 | 1.58 (1.27, 1.97) | 0.002 |
| BMI Z- score | 1.54(1.42,1.68) | <0.001 | 1.59 (1.44, 1.75) | <0.001 | 1.51 (1.24, 1.85) | 0.002 |

Model 1: unadjusted model; Model 2: gender, age, Race/ethnicity, Marital status; Model 3: gender, age, Race/ethnicity, Marital status, Physical Activity, Alcohol, Smoking, Total water intake, Total carbohydrate intake, Total Fat intake, Total protein intake, Hypertension, Diabetes, Cancer, CVD. Abbreviations: ABSI, A Body Shape Index; BMI, body mass index; CI, conicity index; WC, waist circumference; WT, weight; WtHR, waist-to-height ratio; BRI, body roundness index; AVI, Abdominal volume index; WWI, Weight-adjusted waist index.
